# Ultrasound guidance and risk for intravascular catheter-related infections among peripheral arterial catheters: a post-hoc analysis of two large randomized-controlled trials

**DOI:** 10.1186/s13613-020-00705-4

**Published:** 2020-07-08

**Authors:** Niccolò Buetti, Stéphane Ruckly, Jean-Christophe Lucet, Lila Bouadma, Carole Schwebel, Olivier Mimoz, Jean-François Timsit

**Affiliations:** 1 University of Paris, INSERM IAME, U1137, Team DeSCID, Paris, France; 2 AP-HP, Infection Control Unit, Bichat- Claude Bernard University Hospital, 46 Rue Henri Huchard, 75877 Paris Cedex, France; 3 Medical and Infectious Diseases Intensive Care Unit, AP-HP, Bichat-Claude Bernard University Hospital, 46 Rue Henri Huchard, 75877 Paris Cedex, France; 4 Medical Intensive Care Unit, Grenoble University Hospital, Grenoble 1 University, La Tronche, France; 5 Services Des Urgences Adultes and SAMU 86, Centre Hospitalier Universitaire de Poitiers, 86021 Poitiers, France; 6 Université de Poitiers, Poitiers, France; 7 Inserm U1070, Poitiers, France; 8 INSERM UMR S 1039, Radiopharmaceutiques Biocliniques, Faculté de Médecine de Grenoble, Domaine de la Merci, 38700 La Tronche, France

**Keywords:** Catheter, Intravascular, Arterial catheter, Catheter tip, Ultrasound, Ultrasound guidance, Arterial, Catheter-related bloodstream infection, infectious risk

## Abstract

**Background:**

The impact on infectious risk of ultrasound guidance at insertion remains controversial in short-term arterial catheters (ACs). The present study investigated the association between ultrasound guidance (US) during AC insertion and major catheter-related infections (MCRI), catheter-related bloodstream infections (CR-BSI) or colonization, using univariate and multivariate marginal Cox model for clustered data. The skin colonization at catheter removal was evaluated to explain our results.

**Results:**

We used individual data from two multicenter randomized-controlled trials (RCTs) that included a total of 3029 patients, 10 ICUs and 3950 ACs. US guidance was used for 386 (9.8%) catheter placements. In the univariate Cox model analysis, AC insertion with US versus without US exhibited similar risks for MCRI (HR 0.86, CI 95% 0.27–2.72, *p* = 0.79), CR-BSI (HR 0.87, CI 95% 0.20–3.72, *p* = 0.85) and catheter colonization (HR 1.31, CI 95% 0.92–1.86, *p* = 0.13). After adjustment on confounders, risks associated with US guidance remained similar *versus* non-US for MCRI (HR 0.71, CI 95% 0.23–2.24, *p* = 0.56), CR-BSI (HR 0.71, CI 95% 0.17–3.00, *p* = 0.63) and catheter colonization (HR 0.92, CI 95% 0.63–1.34, *p* = 0.67). No differences between US and non-US for MCRI, CR-BSI and colonization were observed according to the insertion site, radial or femoral. At catheter removal, the skin colonization was similar between US and non-US groups (*p* = 0.69).

**Conclusions:**

Using the largest dataset ever collected from large multi-centric RCTs conducted with relatively consistent insertion and maintenance catheter protocols, we showed that the risk of infectious complications for ACs inserted under US guidance is not superior compared to those inserted without US guidance.

*Trial registration* These studies were registered within ClinicalTrials.gov (numbers NCT01629550 and NCT 01189682).

## Background

Arterial catheters (ACs) are instrumental for managing critically ill patients, to facilitate hemodynamic monitoring and frequent blood sampling [1]. To date, the duration of catheter maintenance of ACs is similar to that of central venous catheters, thus now associated with a substantial infection risk [2]. Traditionally, anatomical ‘landmarks’ on the body surface were used to find the correct place in which to insert catheters. However, arterial catheterization using anatomical ‘landmarks’ is associated with an increase in the number of attempts and time needed for successful cannulation [3–5]. The current literature shows that ultrasound imaging may offer gains in safety and quality compared with an anatomical landmark technique. However, the ultrasound guidance effect on infectious risk remains controversial. Indeed, the ultrasound may have its own infectious risk. To our knowledge, no large randomized-controlled trials (RCTs) analyzed the infectious risk between both AC insertion strategies, anatomical landmarks’ technique and ultrasound guidance. Our primary aim was to investigate the association between ultrasound guidance (US) for AC insertion and the intravascular catheter-related infection or colonization, using data gathered for two large RCTs with an extensive prospective data collection at catheter insertion and catheter removal [6, 7].

## Methods

### Design

We used the data from two large RCTs that investigated various prevention strategies, and for which an extensive prospective data collection at catheter insertion and catheter removal was performed [6, 7].

### Patients and setting

Patients were recruited from 2011 to 2014 in various intensive care units (ICUs) in France as soon as they required a catheterization with a short-term central venous catheter (CVC) or a peripheral AC with an expected duration of use of more than 48 h.

### Catheters

For the current study we included only data related to AC. All study centers complied with the French recommendations for catheter insertion and care, which are similar to CDC recommendations [8]: (1) maximal sterile barrier precautions (large sterile drape; surgical hand antisepsis; and mask, cap, sterile gloves, and gown); (2) the site of insertion was left to the discretion of the physician caring for the patient; (3) alcoholic povidone iodine solution or chlorhexidine gluconate was used for skin antisepsis at catheter insertion and during dressing changes; (4) semipermeable chlorhexidine-impregnated or standard dressing was used at all insertion sites and was changed 24 h after catheter insertion and then every 3 or 7 days according to standard practice in each ICU. Leaking, soiled, or wet dressings were changed immediately. Ultrasound guidance was used at the discretion of the attending physician and this variable was routinely collected. A transducer with a sterile sheath was used to perform vascular access procedures. Sterile gel was used. Antiseptic- or antibiotic-impregnated ACs were not used in any of the study ICUs. A check list was routinely used. The catheters were removed if unnecessary or if an infection was suspected. The patients underwent follow-up until 48 h after ICU discharge.

### Definitions and outcomes

According to French and American guidelines, the following definitions were used [9, 10]. Catheter colonization was defined as a quantitative catheter tip culture yielding ≥ 1000 colony-forming units (cfu)/mL. A catheter-related clinical sepsis without bloodstream infection (BSI) was a combination of body temperature (≥ 38.5 °C or ≤ 36.5 °C); catheter colonization; presence of pus at the insertion site or resolution of clinical sepsis after catheter removal; and the absence of any other infectious focus. A catheter-related bloodstream infection (CR-BSI) was a combination of (1) one or more positive peripheral blood cultures sampled 48 h before or after catheter removal; (2) the isolation of the same organism from the colonized catheter or from the catheter insertion site, or a blood culture differential time to positivity of 2 h or more [11]; and (3) no apparent source of bacteremia other than the catheter. If a patient had a positive blood culture for coagulase-negative staphylococci (CoNS), the same pulsotype from the strains recovered from the catheter and blood culture was required for a diagnosis of CR-BSI. A major catheter-related infection (MCRI) was defined as either a catheter-related clinical sepsis without BSI, or a CR-BSI. For patients without any catheter cultures, a blinded adjudication committee determined whether a MCRI was present; sepsis or BSI were classified as catheter-related when there was no other detectable cause of sepsis with or without BSI. The skin colonization was evaluated using semi-quantitative insertion-site cultures: the insertion site was sampled immediately before catheter removal. Because the size of the counting surface was different across studies, we created a semi-quantitative variable with sterile (i.e., negative quantitative cultures), low-grade skin colonization, and high-grade skin colonization according to the median of quantitative cultures obtained in each study.

### Statistical analysis

Characteristics of patients and catheters were described as count (percent) or median (interquartile range) for qualitative and quantitative variables, respectively, and were compared between catheters groups using Chi square, Fisher or Mann–Whitney tests, as appropriate.

The statistical plan had two objectives: (1) to identify the risk differences in MCRI, CR-BSI and catheter colonization between catheters inserted using US guidance and catheter inserted with using anatomical ‘landmarks’ (non-US); (2) to perform a confirmatory analysis analyzing differences in skin colonization at removal between US and non-US.

For the first objective, we used a marginal Cox model for clustered data (PROC PHREG of SAS), to take into account a possible clustering effect of multiple catheters per patient. This model takes into account the censored nature of the data and possible intra-cluster dependence using a robust sandwich covariate estimate. Analyses were stratified by ICU and data were censored at 28 days since catheter insertion. Hazard risk for MCRI, CR-BSI and catheter colonization was evaluated by univariate and multivariate analysis. The variable “ultrasound guidance” (US *vs.* non-US) was forced in our multivariate models and the other variables showing significance in the univariate analysis were used as adjustment factors. The choice of adjustment variables was based on the results of the univariate analysis and refined by including clinically relevant variables (i.e., Simplified Acute Physiology Score [SAPS] II score). The proportionality of hazard risks for catheter type was tested using Martingale residuals.

Confirmatory subgroup analyses were performed according to the insertion site (radial and femoral) and the duration of catheter maintenance (≤ 7 days and > 7 days). Tests were two-tailed, with *p* < 0.05 being considered significant. All analyses were performed using SAS (version 9.4; SAS Institute, Cary, NC). All studies were approved by the national ethics committee.

## Results

### Patients and catheters

**Fig. 1:**
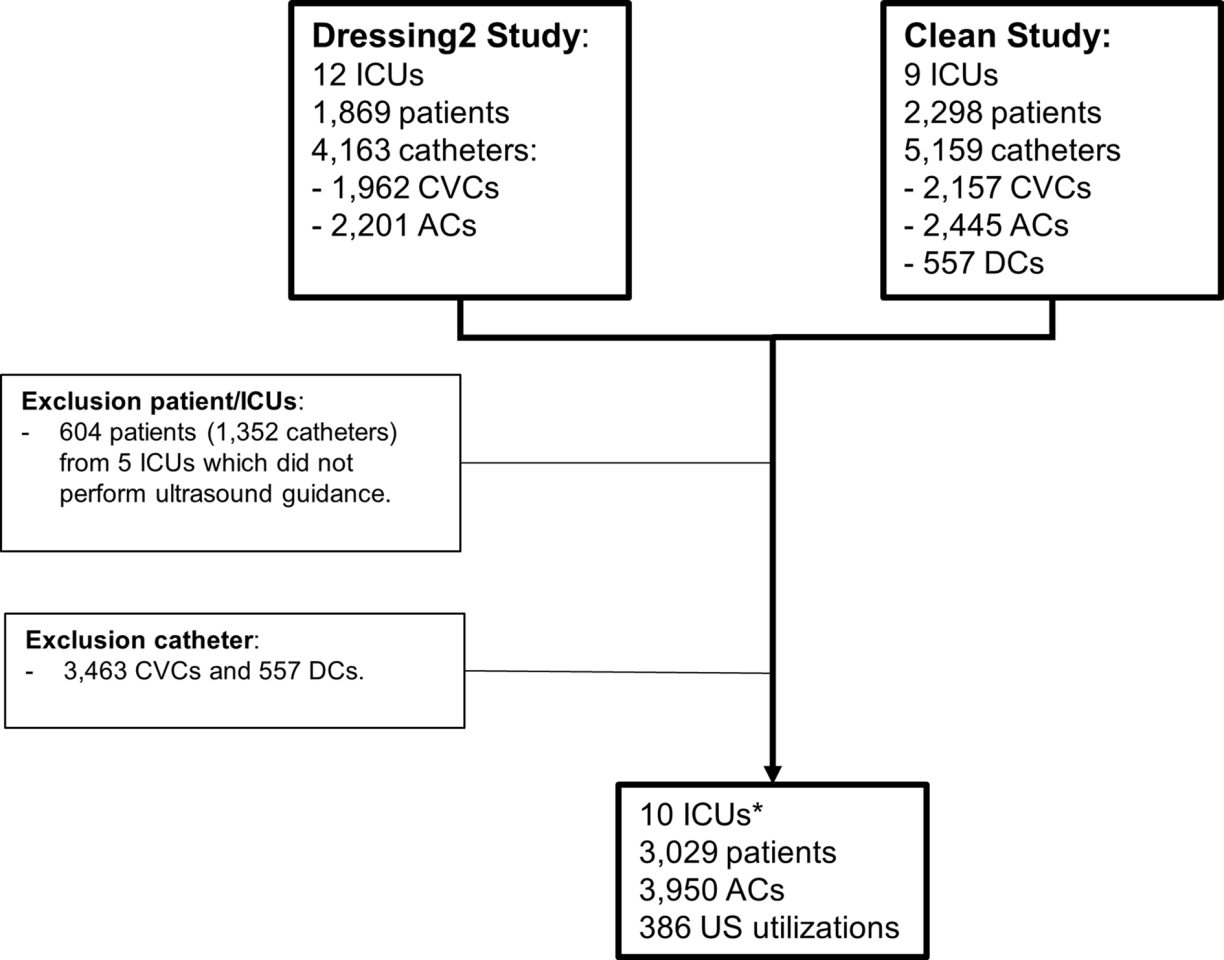
Flow chart. *ICU* intensive care unit, *CVC* central venous catheter, *AC* arterial catheter, *DC* dialysis catheter, *US* ultrasound guidance. *6 ICUs were included in both studies

A total of 3029 patients were included by 10 ICUs in this study and 3950 ACs were analyzed (Fig. 1).

The US was used for 386 catheter placements (356 different patients). Characteristics of the patients and catheters are described in Tables 1 and 2. In the US group, the patients were more frequently admitted for shock (48%) and their SAPS II at study inclusion was significantly higher (54, IQR [41; 68]) than that of patients without US.

**Table 1:** Patients’ characteristics

	Non-US (*n* = 2673)	US (*n* = 356)	*p* value
Sex			
Female	934 (34.9)	137 (38.5)	0.19
Male	1739 (65.1)	219 (61.5)	
Age, median (IQR)	63 [52; 74]	63 [52.5; 73]	0.98
Reason for ICU admission			
Renal failure	113 (4.2)	17 (4.8)	< 0.01
Shock	920 (34.4)	171 (48)	
Coma	265 (9.9)	19 (5.3)	
Other	362 (13.5)	46 (12.9)	
Respiratory failure	811 (30.3)	78 (21.9)	
Trauma	202 (7.6)	25 (7)	
No comorbidity	1803 (67.5)	242 (68)	0.84
Chronic renal failure	101 (3.8)	15 (4.2)	0.69
Chronic cardiac failure	154 (5.8)	24 (6.7)	0.46
Diabetes mellitus	175 (6.5)	21 (5.9)	0.64
Chronic respiratory failure	153 (5.7)	11 (3.1)	0.04
Immunosuppression	234 (8.8)	36 (10.1)	0.40
Hematologic neoplasia	119 (4.5)	13 (3.7)	0.49
MV at admission	1912 (71.5)	268 (75.3)	0.14
Vasopressor at admission	1067 (39.9)	99 (27.8)	< 0.01
SAPS II score, median (IQR)	50 [37; 65]	54 [41; 68]	0.02

*IQR* interquartile range, *ICU* intensive care unit, *MV* mechanical ventilation, *SAPS II score* simplified Acute Physiology Score II. In 30 patients, the ultrasound guidance was used > 1 time

**Table 2:** Catheters’ characteristics

	Non-US (n = 3564)	US (n = 386)	p-value
Catheter days, median (IQR)	5 [2, 9]	5 [2, 9]	0.65
Experience of the operator			
< 50 procedures	2131 (59.8)	269 (69.7)	< 0.01
≥ 50 procedures	1433 (40.2)	117 (30.3)	
Insertion site			
Femoral	1081 (30.3)	226 (58.5)	< 0.01
Radial	2483 (69.7)	160 (41.5)	
Dressing			
CHG-impregnated	763 (21.4)	11 (2.8)	< 0.01
Standard dressing	2801 (78.6)	375 (97.2)	
Skin antisepsis			
Not CHG	1482 (41.6)	188 (48.7)	< 0.01
CHG	2082 (58.4)	198 (51.3)	
MV at insertion	2467 (69.2)	280 (72.5)	0.18
Vasopressor at insertion	1471 (41.3)	223 (57.8)	< 0.01
Antibiotic at insertion	1933 (54.2)	235 (60.9)	0.01
MCRI	29 (0.8)	3 (0.8)	0.94
CR-BSI	19 (0.5)	2 (0.5)	0.97
Colonization	269 (7.5)	38 (9.8)	0.11

*IQR* interquartile range, *ICU* intensive care unit, *MV* mechanical ventilation, *SAPS II score* Simplified Acute Physiology Score II, *CHG* chlorhexidine gluconate, *MCRI* major catheter-related infection, *CR-BSI* catheter-related bloodstream infection

US was more frequently used by junior operators (70%) and for the femoral site insertion (59%). In addition, the skin antisepsis and the dressing used were different according to the ultrasound utilization. We observed 32 MCRIs, 21 CR-BSI and 307 colonizations.

### Catheter infections and colonizations

In the univariate Cox model analysis, the risk for MCRI (HR 0.86, CI 95% 0.27–2.72, *p* = 0.79), CR-BSI (HR 0.87, CI 95% 0.20–3.72, *p* = 0.85) and catheter colonization (HR 1.31, CI 95% 0.92–1.86, *p* = 0.13) was similar for ACs in the US group compared to those of the non-US group (Additional file 1: Tables S1–S3). The proportionality of hazard was respected for MCRI, CR-BSI and colonization.

**Fig. 2:**
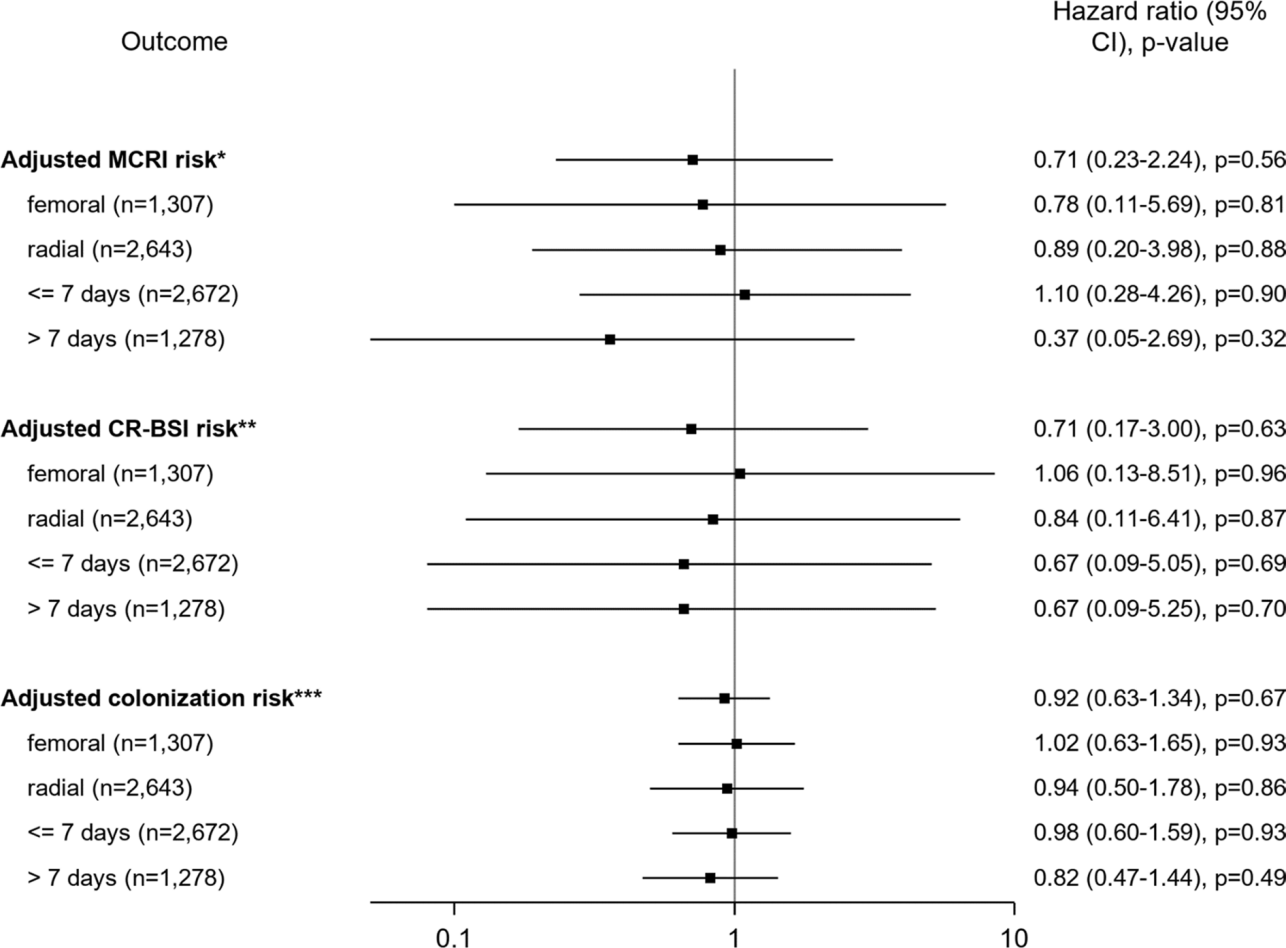
Adjusted analyses for risk of MCRI, CR-BSI and colonization for ultrasound guidance *versus* without ultrasound guidance. *Variables used for adjusting MCRI: SAPS II score, dressing, skin antisepsis, vasopressor at insertion. **Variables used for adjusting CR-BSI: SAPS II score, skin antisepsis and antibiotic at insertion. ***Variables used for adjusting colonization: vasopressor at admission, SAPS II score, insertion site, dressing, skin antisepsis, mechanical ventilation at insertion, vasopressor at insertion, and antibiotics at insertion. A hazard ratio (HR) less than one indicated a lower risk of event of ultrasound guidance (US) compared with non-US. *CI* confidence interval, *MCRI* Major catheter-related infection, *CR-BSI* catheter-related bloodstream infection

In multivariate marginal Cox model, US showed similar risk compared to non-US for MCRI (HR 0.71, CI 95% 0.23–2.24, *p* = 0.56), CR-BSI (HR 0.71, CI 95% 0.17–3.00, *p* = 0.63) and catheter colonization (HR 0.92, CI 95% 0.63–1.34, *p* = 0.67, Fig. 2 and Additional file 1: Tables S1–S3).

Variables independently associated with MCRI (Additional file 1: Table S1) were non-chlorhexidine skin antisepsis (HR 6.42, 95% CI 2.58–15.98, *p* < 0.01) and vasopressor at insertion (HR 0.49, 95% CI 0.23–1.05, *p* = 0.07). Variables independently associated with CR-BSI (Additional file 1: Table S2) were non-chlorhexidine skin antisepsis (HR 6.07, 95% CI 2.2–16.74, *p* < 0.01) and antibiotics at insertion (HR 0.34, 95% CI 0.13–0.87, *p* = 0.02). Variables independently associated with colonization (Additional file 1: Table S3) were vasopressor at admission (HR 0.78, 95% CI 0.60–0.99, *p* = 0.05), femoral insertion site (HR 1.50; 95% CI 1.18–1.92, *p* < 0.01), standard dressing (HR 2.53, 95% CI 1.59–4.03, *p* < 0.01), non-chlorhexidine skin disinfection (HR 6.0, 95% CI 4.49–8.01, *p* < 0.01), mechanical ventilation at insertion (HR 0.80, 95% CI 0.62–1.03, *p* = 0.08) and antibiotics at insertion (HR 0.56, 95% CI 0.44–0.70, *p* < 0.01).

### Confirmatory analyses

Among femoral catheters (*n* = 1307), no differences between US and non-US for MCRI, CR-BSI and colonization were observed (Fig. 2). Similarly, for radial catheters (= 2643) a similar risk for MCRI, CR-BSI and colonization was showed. In the subgroup analysis including only catheters with ≤ 7 days maintenance (*n* = 2672), the MCRI, CR-BSI and colonization risk for US did not differ from non-US.

The skin colonization at catheter removal was similar between US and non-US groups (*p* = 0.69, Table 3). No difference was observed in the different subgroups.

**Table 3:** Skin colonization at catheter removal (main group and subgroup analyses)

	No US	US	*p* value*
All			
High-grade colonization	886 (34.5)	108 (36.9)	0.69
Low-grade colonization	865 (33.6)	93 (31.7)	
Sterile	820 (31.9)	92 (31.4)	
Femoral			
High-grade colonization	268 (37.1)	57 (35.4)	0.84
Low-grade colonization	243 (33.6)	58 (36)	
Sterile	212 (29.3)	46 (28.6)	
Radial			
High-grade colonization	618 (33.4)	51 (38.6)	0.22
Low-grade colonization	622 (33.7)	35 (26.5)	
Sterile	608 (32.9)	46 (34.8)	
≤ 7 days			
High-grade colonization	506 (29.2)	69 (34.5)	0.30
Low-grade colonization	624 (36)	66 (33)	
Sterile	601 (34.7)	65 (32.5)	
> 7 days			
High-grade colonization	380 (45.2)	39 (41.9)	0.78
Low-grade colonization	241 (28.7)	27 (29)	
Sterile	219 (26.1)	27 (29)	

Skin cultures at removal were not performed for 1086 catheters. *Chi square test was performed

## Discussion

Using prospectively collected data from two RCTs, we showed that the US at AC insertion did not influence the risk of intravascular catheter infections. Data in the literature about the role of US in intravascular AC infections are scarce. The current literature mostly focus only on first-attempt failure, mean attempts to success, mean time to success, and the occurrence of hematoma or venipuncture complications [3–5]. Interestingly, all RCTs investigating the role of US for the radial artery in adults assessed only non-infectious complications, thus disregarding intravascular catheter infections [12–17]. Similarly, among femoral artery cannulations, all RCTs were performed in cardiologic patients and mainly focused on short-term complications without considering catheter infections [18–21]. To date, the role of US in the context of intravascular arterial catheter infection is not clear. The use of an additional device, such as ultrasound, may complicate AC insertion and set the stage for breaches in aseptic non-touch technique, and the gel used for optimizing visibility may increase the risk of catheter infection [22, 23]. However, a shorter insertion time and fewer insertion attempts may counterbalance the risk for infection. In an environment of consistent catheter care representing the largest dataset ever assembled, we showed that the US did not increase the infectious risk among ACs. No differences were observed according to the body site of insertion, femoral or radial, and the skin colonization at removal was similar between the two groups. Therefore, our results support the growing evidence that recommends using US routinely [1, 3–5].

Our study has several limitations. First, its design is observational and the US utilization was not randomized. However, we presented exhaustive data that have been prospectively collected by trained investigators and study monitors during RCTs, and we adjusted our analyses on major confounders. Second, no data on the US type (e.g., Doppler *vs*. ultrasound guidance) or sterile sheath used were included. Third, all RCTs were conducted in University-affiliated ICUs in France from 2011 to 2014, thus limiting the generalizability of the results. However, aseptic insertion techniques have not been changed since 2014. Fourth, a post-hoc analysis of our dataset showed a 22.5% probability that US was associated with a falsely significant increased HR of ≥ 1.1 for MCRI (18% for colonization). Fifth, no information on the number of attempts and other mechanical complications were included. However, these outcomes were exhaustively explored in previous analyses. Sixth, catheter cultures were not performed in 406 catheters. However, the rate of missing information was not different between both groups. Finally, we described a large database designed to investigate the impact of certain prevention measures, and interactions may have occurred among the various study groups. However, our statistical analyses considered these potential drawbacks.

## Conclusion

Using the largest dataset ever collected from large multi-centric RCTs conducted with relatively consistent insertion and maintenance catheter protocols, we showed that the risk of infectious complications for arterial catheters inserted under US guidance is not superior compared to those inserted without US guidance. Large RCTs primarily designed to investigate the true impact of US guidance on the infectious risk of arterial catheters are warranted.

## Supplementary information

**Additional file 1.** Univariate and multivariate Cox models for MCRI, CR-BSI and colonization.

## Data Availability

The datasets used and/or analyzed during the current study are available from the corresponding author upon reasonable request.

## Abbreviations

AC: Arterial catheter
BSI: Bloodstream infection
CI: Confidence interval
CVC: Central venous catheter
CR-BSI: Catheter-related Bloodstream Infection
CoNS: Coagulase-negative staphylococci
HR: Hazard ratio
ICU: Intensive Care Unit
MCRI: Major Catheter-related Infection
RCT: Randomized-controlled trial
SAPS: Simplified Acute Physiology Score
US: Ultrasound guidance
