# Supplementary material for: Ultrasound guidance and risk for intravascular catheter-related infections among peripheral arterial catheters: a post-hoc analysis of two large randomized-controlled trials
Source: Ann Intensive Care. 2020 Jul 8;10:89. doi: 10.1186/s13613-020-00705-4 (PMC7343671; doi:10.1186/s13613-020-00705-4)
Supplement: Supplementary file 1 — Additional file 1. Univariate and multivariate Cox models for MCRI, CR-BSI and colonization. [file 13613_2020_705_MOESM1_ESM.docx]

**Additional files**

**Table S1: Univariate and multivariate Cox models for MCRI.**

|  |  | No MCRI | MCRI | HR | 95% CI | p-value | HR | 95% CI | p-value |
| --- | --- | --- | --- | --- | --- | --- | --- | --- | --- |
|  |  |  |  | **Univariate** |  |  | **Multivariate** |  |  |
| US |  | **383 (9.8)** | **3 (9.4)** | **0.86** | **[0.27-2.72]** | **0.790** | **0.71** | **[0.23-2.24]** | **0.561** |
| Sex | Female | 1399 (35.7) | 13 (40.6) | 1.45 | [0.7-3.03] | 0.319 |  |  |  |
|  | Male | 2519 (64.3) | 19 (59.4) |  |  |  |  |  |  |
| Age, median (IQR) | | 63 [52 ; 73] | 60.5 [48.5 ; 66] | 0.99 | [0.97-1.01] |  |  |  |  |
| Reason for ICU admission | Shock | 1385 (35.3) | 8 (25) | 0.40 | [0.13-1.2] |  |  |  |  |
|  | Coma | 353 (9) | 4 (12.5) | 1.13 | [0.32-4.02] |  |  |  |  |
|  | Other | 700 (17.9) | 4 (12.5) | 0.41 | [0.12-1.48] |  |  |  |  |
|  | Respiratory failure | 1174 (30) | 8 (25) | 0.44 | [0.15-1.28] |  |  |  |  |
|  | Trauma | 306 (7.8) | 8 (25) |  |  | 0.227 |  |  |  |
| No comorbidity | | 2670 (68.1) | 23 (71.9) | 1.13 | [0.5-2.57] | 0.765 |  |  |  |
| Chronic heart failure | | 248 (6.3) | 3 (9.4) | 1.28 | [0.29-5.7] | 0.743 |  |  |  |
| Diabetes mellitus | | 250 (6.4) | 4 (12.5) | 1.98 | [0.72-5.46] | 0.186 |  |  |  |
| Chronic respiratory failure | | 213 (5.4) | 1 (3.1) | 0.82 | [0.11-5.96] | 0.843 |  |  |  |
| Immunosuppression | | 334 (8.5) | 2 (6.3) | 0.74 | [0.18-3.1] | 0.685 |  |  |  |
| MV at admission | | 2888 (73.7) | 26 (81.3) | 1.05 | [0.44-2.49] | 0.908 |  |  |  |
| Vasopressor at admission | | 2498 (63.8) | 18 (56.3) | 0.62 | [0.3-1.28] | 0.197 |  |  |  |
| SAPS II score, median (IQR) | | 51 [38 ; 66] | 47 [26.5 ; 65] | 0.99 | [0.97-1.01] | 0.200 | 0.99 | [0.97-1.01] | 0.345 |
| ICU mortality | | 1235 (31.5) | 14 (43.8) | 1.57 | [0.76-3.24] | 0.221 |  |  |  |
| Hospital mortality | | 1460 (37.3) | 15 (46.9) | 1.33 | [0.65-2.73] | 0.439 |  |  |  |
| LOS hospital, median (IQR) | | 28 [13 ; 53] | 37 [20 ; 55] | 0.995 | [0.98-1.01] | 0.432 |  |  |  |
| Time between ICU admission and catheter insertion, days median (IQR) | | 1 [1 ; 5] | 2.5 [1 ; 12] | 0.99 | [0.95-1.03] | 0.655 |  |  |  |
| Experience of the operator | < 50 procedures | 2381 (60.8) | 19 (59.4) | 0.91 | [0.44-1.91] | 0.804 |  |  |  |
|  | ≥ 50 procedures | 1537 (39.2) | 13 (40.6) |  |  |  |  |  |  |
| Insertion site | Femoral | 1298 (33.1) | 9 (28.1) | 0.94 | [0.45-1.95] | 0.861 |  |  |  |
|  | Radial | 2620 (66.9) | 23 (71.9) |  |  |  |  |  |  |
| Dressing | CHG-impregnated | **772 (19.7)** | **2 (6.3)** | **0.23** | **[0.05-0.98]** | **0.047** |  |  |  |
|  | Standard | **3146 (80.3)** | **30 (93.8)** |  |  |  | 2.52 | [0.57-11.14] | 0.223 |
| Skin antisepsis | Non CHG | **1644 (42)** | **26 (81.3)** | **7.32** | **[3.05-17.57]** | **<0.001** | **6.42** | **[2.58-15.98]** | **<0.001** |
|  | CHG | **2274 (58)** | **6 (18.8)** |  |  |  |  |  |  |
| MV at insertion | | 2725 (69.6) | 22 (68.8) | 0.62 | [0.31-1.23] | 0.173 |  |  |  |
| Vasopressor at insertion | | **1686 (43)** | **8 (25)** | **0.42** | **[0.2-0.9]** | **0.025** | 0.49 | [0.23-1.05] | 0.068 |
| Antibiotics at insertion | | 2152 (54.9) | 16 (50) | 0.59 | [0.29-1.22] | 0.156 |  |  |  |

Legends. IQR: Interquartile range. CI: Confidence interval. HR: hazard ratio. US: Ultrasound guidance. ICU: Intensive care unit. MV: Mechanical ventilation. SAPS II score: Simplified Acute Physiology Score II. LOS: length of hospital stay. CHG: chlorhexidine-gluconate. MCRI: Major catheter-related infection. CR-BSI: Catheter-related bloodstream infection. A sensitivity analysis excluding CHG-impregnated dressings showed similar results (adjusted HR 0.714, CI 95% 0.23 – 2.24, p=0.564)

**Table S2: Univariate and multivariate Cox models for CR-BSI.**

|  |  | No CR-BSI | CR-BSI | HR | 95% CI | p-value | HR | 95% CI | p-value |
| --- | --- | --- | --- | --- | --- | --- | --- | --- | --- |
|  |  |  |  | **Univariate** |  |  | **Multivariate** |  |  |
| US |  | **384 (9.8)** | **2 (9.5)** | **0.87** | **[0.2-3.72]** | **0.846** | **0.71** | **[0.17-3]** | **0.636** |
| Sex | Female | 1404 (35.7) | 8 (38.1) | 1.36 | [0.56-3.33] | 0.497 |  |  |  |
|  | Male | 2525 (64.3) | 13 (61.9) |  |  |  |  |  |  |
| Age, median (IQR) | | 63 [52 ; 73] | 62 [48 ; 66] | 0.99 | [0.96-1.02] | 0.448 |  |  |  |
| Reason for ICU admission | Shock | 1388 (35.3) | 5 (23.8) | 0.51 | [0.12-2.15] |  |  |  |  |
|  | Coma | 354 (9) | 3 (14.3) | 1.86 | [0.44-7.83] |  |  |  |  |
|  | Other | 700 (17.8) | 4 (19) | 0.91 | [0.24-3.5] |  |  |  |  |
|  | Respiratory failure | 1177 (30) | 5 (23.8) | 0.58 | [0.16-2.12] |  |  |  |  |
|  | Trauma | 310 (7.9) | 4 (19) |  |  | 0.431 |  |  |  |
| No comorbidity | | 2678 (68.2) | 15 (71.4) | 1.12 | [0.4-3.16] | 0.830 |  |  |  |
| Chronic heart failure | | 249 (6.3) | 2 (9.5) | 1.24 | [0.17-9.06] | 0.833 |  |  |  |
| Diabetes mellitus | | 252 (6.4) | 2 (9.5) | 1.48 | [0.36-6.11] | 0.588 |  |  |  |
| Chronic respiratory failure | | 213 (5.4) | 1 (4.8) | 1.33 | [0.19-9.49] | 0.778 |  |  |  |
| Immunosuppression | | 335 (8.5) | 1 (4.8) | 0.562 | [0.08-4.13] | 0.571 |  |  |  |
| MV at admission | | 2897 (73.7) | 17 (81) | 1.01 | [0.35-2.93] | 0.991 |  |  |  |
| Vasopressor at admission | | 2503 (63.7) | 13 (61.9) | 0.78 | [0.32-1.87] | 0.571 |  |  |  |
| SAPS II score, median (IQR) | | 51 [38 ; 66] | 46 [24 ; 67] | 0.98 | [0.95-1.01] | 0.254 | 0.98 | [0.95-1.01] | 0.248 |
| ICU mortality | | 1239 (31.5) | 10 (47.6) | 1.87 | [0.77-4.59] | 0.169 |  |  |  |
| Hospital mortality | | 1465 (37.3) | 10 (47.6) | 1.40 | [0.58-3.43] | 0.455 |  |  |  |
| LOS hospital, median (IQR) | | 28 [13 ; 53] | 33 [20 ; 54] | 0.996 | [0.98-1.01] | 0.644 |  |  |  |
| Time between ICU admission and catheter insertion, days median (IQR) | | 1 [1 ; 5] | 3 [1 ; 12] | 1.00 | [0.95-1.05] | 0.985 |  |  |  |
| Experience of the operator | < 50 procedures | 2386 (60.7) | 14 (66.7) | 1.24 | [0.46-3.35] | 0.666 |  |  |  |
|  | ≥ 50 procedures | 1543 (39.3) | 7 (33.3) |  |  |  |  |  |  |
| Insertion site | Femoral | 1301 (33.1) | 6 (28.6) | 0.95 | [0.38-2.36] | 0.903 |  |  |  |
|  | Radial | 2628 (66.9) | 15 (71.4) |  |  |  |  |  |  |
| Dressing | CHG-impregnated | 773 (19.7) | 1 (4.8) | 0.17 | [0.02-1.28] | 0.085 |  |  |  |
|  | Standard | 3156 (80.3) | 20 (95.2) |  |  |  |  |  |  |
| Skin antisepsis | Non CHG | **1654 (42.1)** | **16 (76.2)** | **5.42** | **[2.01-14.59]** | **<0.001** | **6.07** | **[2.2-16.74]** | **<0.001** |
|  | CHG | **2275 (57.9)** | **5 (23.8)** |  |  |  |  |  |  |
| MV at insertion | | 2732 (69.5) | 15 (71.4) | 0.71 | [0.29-1.73] | 0.448 |  |  |  |
| Vasopressor at insertion | | 1688 (43) | 6 (28.6) | 0.50 | [0.21-1.21] | 0.124 |  |  |  |
| Antibiotics at insertion | | **2160 (55)** | **8 (38.1)** | **0.36** | **[0.15-0.89]** | **0.027** | **0.34** | **[0.13-0.87]** | **0.024** |

Legends. IQR: Interquartile range. CI: Confidence interval. HR: hazard ratio. US: Ultrasound guidance. ICU: Intensive care unit. MV: Mechanical ventilation. SAPS II score: Simplified Acute Physiology Score II. LOS: length of hospital stay. CHG: chlorhexidine-gluconate. CR-BSI: Catheter-related bloodstream infection. CR-BSI: Catheter-related bloodstream infection.

**Table S3: Univariate and multivariate Cox models for colonization.**

|  |  | No colonization | Colonization | HR | 95% CI | p-value | HR | 95% CI | p-value |
| --- | --- | --- | --- | --- | --- | --- | --- | --- | --- |
|  |  |  |  | **Univariate** |  |  | **Multivariate** |  |  |
| US |  | **348 (9.6)** | **38 (12.4)** | **1.31** | **[0.92-1.86]** | **0.131** | **0.92** | **[0.63-1.34]** | **0.670** |
| Sex | Female | 1309 (35.9) | 103 (33.6) | 1.02 | [0.8-1.3] | 0.884 |  |  |  |
|  | Male | 2334 (64.1) | 204 (66.4) |  |  | 0.884 |  |  |  |
| Age, median (IQR) | | 63 [52 ; 73] | 63 [51 ; 73] | 1.00 | [0.99-1.01] | 0.795 |  |  |  |
| Reason for ICU admission | Shock | 1295 (35.5) | 98 (31.9) | 1.14 | [0.72-1.8] | 0.570 |  |  |  |
|  | Coma | 338 (9.3) | 19 (6.2) | 1.00 | [0.5-2.01] | 0.995 |  |  |  |
|  | Other | 644 (17.7) | 60 (19.5) | 1.14 | [0.69-1.87] | 0.615 |  |  |  |
|  | Respiratory failure | 1079 (29.6) | 103 (33.6) | 1.22 | [0.77-1.93] | 0.403 |  |  |  |
|  | Trauma | 287 (7.9) | 27 (8.8) |  |  | 0.910 |  |  |  |
| No comorbidity | | 2481 (68.1) | 212 (69.1) | 1.09 | [0.85-1.39] | 0.514 |  |  |  |
| Chronic heart failure | | 227 (6.2) | 24 (7.8) | 1.13 | [0.76-1.69] | 0.546 |  |  |  |
| Diabetes mellitus | | 234 (6.4) | 20 (6.5) | 0.89 | [0.54-1.47] | 0.651 |  |  |  |
| Chronic respiratory failure | | 192 (5.3) | 22 (7.2) | 1.39 | [0.87-2.2] | 0.166 |  |  |  |
| Immunosuppression | | 310 (8.5) | 26 (8.5) | 0.98 | [0.65-1.47] | 0.919 |  |  |  |
| MV at admission | | 2686 (73.7) | 228 (74.3) | 0.78 | [0.6-1.02] | 0.072 |  |  |  |
| Vasopressor at admission | | **2338 (64.2)** | **178 (58)** | **0.70** | **[0.55-0.89]** | **0.004** | **0.78** | **[0.60-0.99]** | **0.046** |
| SAPS II score, median (IQR) | | **51 [38 ; 66]** | **48 [34 ; 64]** | **0.99** | **[0.99-1]** | **0.036** | 0.99 | [0.99-1.00] | 0.116 |
| ICU mortality | | 1134 (31.1) | 115 (37.5) | 1.23 | [0.97-1.55] | 0.089 |  |  |  |
| Hospital mortality | | 1342 (36.8) | 133 (43.3) | 1.13 | [0.89-1.42] | 0.311 |  |  |  |
| LOS hospital, median (IQR) | | 27 [13 ; 51] | 37 [20 ; 72] | 0.996 | [0.99-1] | 0.012 |  |  |  |
| Time between ICU admission and catheter insertion, days median (IQR) | | 1 [1 ; 5] | 2 [1 ; 14] | 1.00 | [0.99-1.01] | 0.711 |  |  |  |
| Experience of the operator | < 50 procedures | 2198 (60.3) | 202 (65.8) | 1.23 | [0.97-1.55] | 0.088 |  |  |  |
|  | ≥ 50 procedures | 1445 (39.7) | 105 (34.2) |  |  | 0.088 |  |  |  |
| Insertion site | Femoral | **1190 (32.7)** | **117 (38.1)** | **1.34** | **[1.06-1.69]** | **0.013** | **1.50** | **[1.18-1.92]** | **0.001** |
|  | Radial | **2453 (67.3)** | **190 (61.9)** |  |  | **0.013** |  |  |  |
| Dressing | CHG-impregnated | **753 (20.7)** | **21 (6.8)** | **0.29** | **[0.19-0.46]** | **<0.001** |  |  |  |
|  | Standard | **2890 (79.3)** | **286 (93.2)** |  |  | **<0.001** | **2.53** | **[1.59-4.03]** | **<0.001** |
| Skin antisepsis | Non CHG | **1423 (39.1)** | **247 (80.5)** | **6.08** | **[4.57-8.09]** | **<0.001** | **6.00** | **[4.49-8.01]** | **<0.001** |
|  | CHG | **2220 (60.9)** | **60 (19.5)** |  |  | **<0.001** |  |  |  |
| MV at insertion | | **2528 (69.4)** | **219 (71.3)** | **0.76** | **[0.59-0.97]** | **0.028** | 0.80 | [0.62-1.03] | 0.082 |
| Vasopressor at insertion | | **1587 (43.6)** | **107 (34.9)** | **0.72** | **[0.57-0.91]** | **0.007** | 0.89 | [0.68-1.15] | 0.363 |
| Antibiotics at insertion | | **2013 (55.3)** | **155 (50.5)** | **0.64** | **[0.52-0.8]** | **<0.001** | **0.56** | **[0.44-0.70]** | **<0.001** |

Legends. IQR: Interquartile range. CI: Confidence interval. HR: hazard ratio. US: Ultrasound guidance. ICU: Intensive care unit. MV: Mechanical ventilation. SAPS II score: Simplified Acute Physiology Score II. LOS: length of hospital stay. CHG: chlorhexidine-gluconate. CR-BSI: Catheter-related bloodstream infection. CR-BSI: Catheter-related bloodstream infection.
